# User-centered design and development of TWIN-Acta: A novel control suite of the TWIN lower limb exoskeleton for the rehabilitation of persons post-stroke

**DOI:** 10.3389/fnins.2022.915707

**Published:** 2022-11-24

**Authors:** Marianna Semprini, Tiziana Lencioni, Wiebke Hinterlang, Christian Vassallo, Silvia Scarpetta, Stefano Maludrottu, Riccardo Iandolo, Marta Carè, Matteo Laffranchi, Michela Chiappalone, Maurizio Ferrarin, Lorenzo De Michieli, Johanna Jonsdottir

**Affiliations:** ^1^Rehab Technologies Lab, Istituto Italiano di Tecnologia, Genoa, Italy; ^2^Department of Informatics, Bioengineering, Robotics, and Systems Engineering (DIBRIS), Universitá degli Studi di Genova, Genoa, Italy; ^3^IRCCS Fondazione Don Carlo Gnocchi, Milan, Italy

**Keywords:** balance, gait, lower limb exoskeleton, neurorehabilitation, rehabilitation, stroke

## Abstract

**Introduction:**

Difficulties faced while walking are common symptoms after stroke, significantly reducing the quality of life. Walking recovery is therefore one of the main priorities of rehabilitation. Wearable powered exoskeletons have been developed to provide lower limb assistance and enable training for persons with gait impairments by using typical physiological movement patterns. Exoskeletons were originally designed for individuals without any walking capacities, such as subjects with complete spinal cord injuries. Recent systematic reviews suggested that lower limb exoskeletons could be valid tools to restore independent walking in subjects with residual motor function, such as persons post-stroke. To ensure that devices meet end-user needs, it is important to understand and incorporate their perspectives. However, only a limited number of studies have followed such an approach in the post-stroke population.

**Methods:**

The aim of the study was to identify the end-users needs and to develop a user-centered-based control system for the TWIN lower limb exoskeleton to provide post-stroke rehabilitation. We thus describe the development and validation, by clinical experts, of TWIN-Acta: a novel control suite for TWIN, specifically designed for persons post-stroke. We detailed the conceived control strategy and developmental phases, and reported evaluation sessions performed on healthy clinical experts and people post-stroke to evaluate TWIN-Acta usability, acceptability, and barriers to usage. At each developmental stage, the clinical experts received a one-day training on the TWIN exoskeleton equipped with the TWIN-Acta control suite. Data on usability, acceptability, and limitations to system usage were collected through questionnaires and semi-structured interviews.

**Results:**

The system received overall good usability and acceptability ratings and resulted in a well-conceived and safe approach. All experts gave excellent ratings regarding the possibility of modulating the assistance provided by the exoskeleton during the movement execution and concluded that the TWIN-Acta would be useful in gait rehabilitation for persons post-stroke. The main limit was the low level of system learnability, attributable to the short-time of usage. This issue can be minimized with prolonged training and must be taken into consideration when planning rehabilitation.

**Discussion:**

This study showed the potential of the novel control suite TWIN-Acta for gait rehabilitation and efficacy studies are the next step in its evaluation process.

## Introduction

Stroke is the second most common cause of disability worldwide, leading to a persistent impairment of lower limb motor functions (Johnson et al., 2016). Hemiparesis is the deficit that occurs most frequently after a stroke event, it affects 80% of people who survive the acute phase, and impairs the lower limb functionality, balance, and gait pattern due to muscle weakness, altered muscle tone, and co-activation of the antagonist’s muscles (Doyle et al., 2010; Hatem et al., 2016). These deficits, which typically affect the lower limb contralateral to the brain hemisphere where the stroke occurred, are the direct consequence of the damage to the cortico-spinal system, which causes difficulties in the transfer of motor commands from the cortex to the spinal cord. It is important to start rehabilitation treatment as soon as the patient appears clinically stable in order to achieve maximum recovery and restore as far as possible the independence of the affected subject (Bernhardt et al., 2017). After recovery and rehabilitation, a large percentage of stroke survivors relearn to walk, but most of them continue to have walking deficits. In such subjects, the pathological gait is characterized by abnormal kinematics and kinetics of both the paretic and non-paretic limbs. These impairments contribute to spatiotemporal asymmetries and gait compensations such as hip hiking and circumduction, ultimately resulting in a slow and metabolically inefficient gait, and in an increased risk of falling. Therefore, there is a need to develop rehabilitative programs to help patients to reduce their impairments and compensations. The amount of walking practice provided during rehabilitation is generally low and the onset is often delayed over time, especially for individuals requiring considerable assistance from their therapist to stand and walk. This clinical practice does not reflect recent guidelines for recovery after stroke, which suggest starting the treatment as soon as possible, already a week after the insult (Bernhardt et al., 2017) and providing constant exercise (Billinger et al., 2014).

Electromechanical devices such as body weight-supported treadmills and treadmill-based robotic devices have been proposed to provide walking practice to non-ambulatory individuals during stroke rehabilitation, though some research has not supported their use (Hsu et al., 2020). Possible reasons for the mixed findings are the suggestion that treadmill-based assisted gait training does not fully replicate the task-specificity of over-ground walking (Rosenblatt and Grabiner, 2010; Hsu et al., 2020) and that body weight support may significantly alter natural joint kinematics (van Hedel et al., 2006; Ferrarin et al., 2018). Furthermore, it must be considered that in the most severe cases in which the functional ability to walk is limited (i.e., Functional Ambulation Category, FAC ranged 1–3), wearable exoskeletons are recommended compared to other systems, such as body weight-supported training (Morone et al., 2017).

The true recovery from stroke impairment is underpinned by spontaneous recovery and neuroplasticity, which reorganizes the brain assigning the functionalities of the injured areas to new ones and/or trying to re-activate the partially injured areas if possible. Neurorobotic devices can boost neuroplasticity and entrain the recovery mechanisms following brain injury with regard to motor performance and gait (Calabrò et al., 2018, 2019).

The volitional participation of the patient in the rehabilitation exercise is an essential component to enhance the functional re-organization of the (central and peripheral) nervous system, which is crucial to regain walking ability after a stroke (Krucoff et al., 2016). In fact, it has been widely demonstrated that a cooperative approach to rehabilitation yields better results and keeps a high level of engagement of the patient during the robotic therapy sessions (Mihelj et al., 2007).

Recent systematic reviews (Mehrholz and Pohl, 2012; Mehrholz et al., 2017), indicated that lower limb exoskeletons are valid tools to help achieve independent walking in persons post-stroke, especially in the first 3 months after stroke. In the last years, many lower-limb exoskeletons presented new assistive features to compensate for asymmetric gait patterns while maintaining some degree of freedom for the patient. The primary goal of exoskeleton-based rehabilitation is to facilitate the recovery of a physiological gait pattern through interaction with the device. It is indeed frequent that patients, although physically able to perform the requested exercise, are hindered in doing so as they have fear of falling. In this context, an exoskeleton is particularly desirable as not only it can automate the walking pattern, but can also provide support to the patient, favoring a confident motor pattern. A comprehensive review of the solutions implemented can be found in Baud et al. (2021). The authors concluded that most control strategies are based on the use of pre-defined trajectories for full-mobilization and event-triggered torque profiles for partial assistance and underlined the need to develop advanced control strategies based on the adaptation of position/torque profiles online during the motor performance (Baud et al., 2021). An example of such an approach is represented by the work of the group Goldfarb, in which an assistive control strategy was applied to the Vanderbilt lower limb exoskeleton for rehabilitation of persons post-stroke (Murray et al., 2014). In that case, the patient rather than the exoskeleton provided movement coordination, without the machine dictating the spatiotemporal nature of the joint movement. More recently, the same group proposed a velocity-based flow field controller for the INDEGO lower limb exoskeleton that provides leg coordination during the swing phase of gait (Martínez et al., 2018). These solutions are indeed preferable for persons post-stroke because they do not impose a predefined gait trajectory and exploit the residual motor ability of the user. Similarly, the HAL lower limb exoskeleton has been used in post-stroke rehabilitation and it was controlled voluntarily by the patient’s own muscle signals detected by surface electrodes (Sczesny-Kaiser et al., 2019). It has also been proposed to gradually drive the user toward a predefined trajectory using a cable-driven active leg exoskeleton (C-ALEX), which allows unrestricted movement and provides continuous force assistance throughout the gait cycle (Hidayah et al., 2020).

Even if exoskeleton-based treatments for post-stroke rehabilitation have great potential, currently available systems mostly consist of cumbersome devices and massive sensor placement over the patient’s body, thus limiting their actual usability in the clinical setting. To this end, more interdisciplinary collaborations between clinicians and technicians are envisaged, to allow the full exploitation of exoskeletons beyond the laboratory space (Baud et al., 2021).

To fill the above technological and clinical gap, we have settled an interdisciplinary group of experts composed of clinicians and technologists aimed at co-designing a new control strategy for the lower limb exoskeleton TWIN (Laffranchi et al., 2021), to be used for the rehabilitation of persons post-stroke.

TWIN is a powered lower-limb exoskeleton developed at the IIT-INAIL Rehab Technologies Lab. It was primarily designed and developed for patients affected by Spinal Cord Injury (SCI). The device was co-designed using a rigorous user-centered approach that involved experts from different backgrounds, who jointly worked to develop a new device for personal use (Laffranchi et al., 2021). Given that patients with SCI have no residual motor function to rely on, the TWIN exoskeleton was initially designed to operate in full position control (Vassallo et al., 2020).

Through this study, we describe the flow of work that was necessary to modify the control strategy of the TWIN-powered lower limb exoskeleton, originally designed for SCI patients (Vassallo et al., 2020; Laffranchi et al., 2021), to allow stroke rehabilitation. Furthermore, we detail the resulting control strategy and show the results of evaluation sessions performed on healthy subjects. In this new control scenario, the goal of the exoskeleton is to restore the physiological gait pattern assisting the motor deficiencies rather than forcing the patient to follow the predefined movement trajectories (Campbell et al., 2020; Baud et al., 2021).

The developmental phase followed a user-centered approach, based on focus group sessions with clinical and biomechanical experts to define the design specifications for the new control modality of the exoskeleton. We specifically focused on the most frequent walking impairments faced by persons post-stroke, namely, increased hip hiking, reduced knee flexion during swing, reduced foot clearance, ankle dorsiflexion, and push-off. Addressing these issues, led to the development of the TWIN-Acta control suite, which enables the TWIN exoskeleton to provide tailored support depending on the patient’s residual skills to boost recovery.

## Materials and methods

The TWIN exoskeleton developed for the assistance of paraplegic subjects was the starting point. As part of the development and testing phase, a series of focus groups and testing studies were carried out with clinical experts. The first version of the TWIN exoskeleton was critically evaluated for its use in the rehabilitation of persons with post-stroke hemiplegia by experienced physiotherapists and engineers working in the rehabilitation field, through an internal focus group (T0) leading to the implementation of new device concepts and control strategies. Three experienced physiotherapists then tested and evaluated four intermediate versions of the exoskeleton (T1) while the final version of *TWIN-Acta*, the exoskeleton control suite specifically adjusted for the training of persons with post-stroke hemiplegia, was tested and evaluated by five internal clinical experts (T2). A panel of four external health professional experts that observed the T2 session provided a further feasibility evaluation of the system.

The final version of the TWIN-Acta was also tested by five persons post-stroke [5 Males, age (yrs) 53.4 ± 9.0, body mass (kg) 77.4 ± 11.0, body height (cm) 173.6 ± 4.2].

The study was conducted in accordance with the Declaration of Helsinki, and approved by the ethical committee of IRCCS Don Carlo Gnocchi Foundation, Milan, Italy (session 21 June 2018) and written informed consent was obtained prior to experimental sessions.

### The TWIN exoskeleton

**FIGURE 1:**
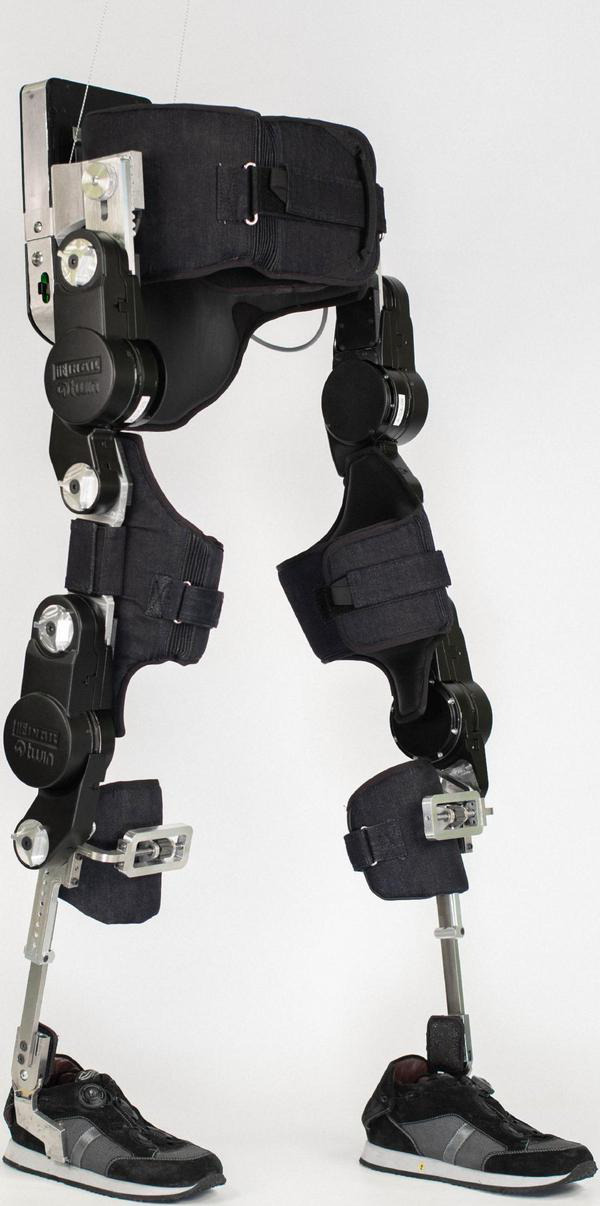
The TWIN lower limb exoskeleton.

The modular mechanical structure of TWIN is shown in Figure 1. It consists of a pelvis module and two legs, which are formed by femur and tibia modules, whose lengths are chosen according to the user’s anthropometric data; all parts are connected by 4 actuated joints at the hip and knee level. The ankle joint is passive and consists of two springs that can be tuned according to the user’s residual skills. Each joint is equipped with a fast-shaft quadrature encoder used for motion control.

The connection between the patient and exoskeleton is achieved through specific fabric braces, while the use of forearm crutches or a rehabilitation walker is mandatory to maintain balance during all locomotion tasks. The ranges of the user’s morphology are height 160–190 cm, max weight 90 kg, thigh length 35.5–47.5 cm, shank length: 40.5–48.5 cm, pelvis width 69–99 cm, shoe size 36–45. The battery pack is located at the back of the device and guarantees up to 3 h of continuous operation. The control of gait parameters can be set using a mobile device-based GUI, whereas each step is triggered by means of an Inertial Measurement Unit (IMU)—based system, placed on the pelvis module.

### Evaluation questionnaires used

Before and after all testing (i.e., wearing the exoskeleton) and observation (i.e., observing another person testing the TWIN-Acta) questionnaires consisting of semi-structured interviews and the System Usability Scale (SUS) (Brooke, 1996) for perceived usability were recorded from experts involved in this study.

The semi-structured interview questions were organized as follows:

• Pre-trial (before testing the TWIN-Acta) questions regarding:
  1. socio-demographic information;
  2. experience of technology used in rehabilitation;
  3. knowledge of exoskeletons.
• Post-trial questions regarded the following aspects of the current state of TWIN-Acta:
  4. positive aspects;
  5. negative aspects;
  6. potential utility of using the system in balance and gait rehabilitation rated on a scale of “not at all useful” (0) to “very useful” (7);
  7. clarity of system functioning;
  8. aspects that could be improved;
  9. usefulness of the system for the rehabilitation of their own patients;
  10. opinion about the willingness of the health institutions to adopt the system for rehabilitation;
  11. suggestions for making TWIN-Acta even more complete as a rehabilitation tool.

The persons post-stroke enrolled tested the exoskeleton operated by the final version of TWIN-Acta in one session, and answered the post-trial questionnaires SUS and the short version SRMS [Stroke Rehabilitation Motivation Scale (White et al., 2012)] scales, both validated for the stroke disease.

After the testing sessions and semi-structured interviews, the overall usability and learnability of the exoskeleton were further evaluated using the modified SUS questionnaire (Brooke, 1996). The SUS comprised items aimed at evaluating whether the system was easy to use, technically consistent, and suitable for continued use in the future. This scale is a widely used generic measure of product usability, whose validity and reliability have been already demonstrated (Borsci et al., 2009). The scale includes the learnability assessment referring to the users’ ability to use the technology. This is a 10-item questionnaire with a 5-point Likert scale, with response options ranging from 1 (Strongly disagree) to 5 (Strongly agree). Items 1, 3, 5, 7, and 9 are positively worded and items 2, 4, 6, 8, and 10 are negatively worded. Using the factor analysis, the SUS is able to provide additional information *via* two sub-scales: an 8-item “Usability” and a 2-item “Learnability” scale (Bangor et al., 2009; Sauro, 2011). The SUS final score and subscores range from 0 to 100, and values >68 are considered acceptable (good usability/learnability). In addition to SUS, persons post-stroke filled out the 7-item SRMS with a 5-point Likert scale in reference to the experience of walking with the TWIN-Acta. The scores for items 1 and 6 were reversed for the calculation of the final SRMS score and ranged from 7 to 35 (higher scores indicated higher motivation). SRMS values equal or greater of 21 were considered normal to high motivation (White et al., 2012).

Expert physical therapists tested the behavior of the exoskeleton with incremental versions of the control software TWIN-Acta during trials of overground walking at a self-selected speed.

### Incremental stages of development and testing

The study began with testing and observation of the TWIN exoskeleton worn by expert engineers and the definition of system requirements for specific use during post-stroke rehabilitation (T0). This first focus group was composed of all authors, combining expert engineers and expert clinicians. It was followed by a developmental phase (T1) composed of four steps in which the intermediate version of the system was tested, respectively, in April 2019 (T1.1), June 2019 (T1.2), November 2019 (T1.3), and January 2020 (T1.4). The final version of *TWIN-Acta*, i.e., the control suite for use of the TWIN exoskeleton in post-stroke rehabilitation, was evaluated in April 2021 (T2) by five health professionals who physically tested the system, and by a focus group of four clinicians who observed the testing sessions. In the following sections, we describe the features of the TWIN-Acta control at each incremental step and the outcomes of its evaluation.

#### Focus group for system requirements and specifications (T0)

This first phase focused on understanding the possible steps to be taken in order to modify the control of the TWIN exoskeleton for neurologic rehabilitation. In order to equip the device with a novel control suite specifically designed for the treatment of persons post-stroke, we first inspected potential strategies that could address the rehabilitative needs of these patients. Specifically, we took into account the following strategies: the *assist*-*as-needed control strategy* and the *implementation of transparent control for the healthy limb*.

A growing number of studies are supporting the evidence that device assistance should be provided according to an *assist-as-needed* approach based on the residual skills of the patients, in order to maximize their learning opportunities and promote neural plasticity (Durandau et al., 2019).

We thus explored possible ways to implement an assist-as-needed control strategy. This requires the design and implementation of control paradigms based on force/torque sensing to implement the user-exoskeleton interaction. Within the context of assist-as-needed control paradigms, we eventually opted for a *free interaction* strategy, which allows the patient to establish the whole gait pattern, with a variable level of joint assistance. This configuration is therefore suitable for chronic or subacute patients with residual voluntary control and may be useful to recovering a correct posture and movement. The full position control mode is preferable for more severe patients with very limited voluntary control. Given the intrinsic asymmetric impairment between sides following stroke, a single leg exoskeleton would ideally suffice for rehabilitation of the paretic side. We therefore implemented and compared three subsequent approaches on healthy subjects:

(1) Use of the exoskeleton with only one physical leg, leaving the other free;
(2) Control of one exoskeleton leg in “transparency mode” (i.e., friction only compensation);
(3) Assistance also to the non-paretic leg to improve the overall stability of the walk (i.e., to favor the knee extension and a correct trunk posture).

Our first attempt was to wear the exoskeleton with only one mechanical leg mounted so that one physical leg (the one simulated as the non-paretic one) was free. However, such a single-leg exoskeleton caused balancing issues leading to instability. The machine was indeed originally designed for two-leg support.

The second approach consisted in wearing the exoskeleton properly mounted but controlling the two legs in two different ways: the paretic side with the assistive mode, and the non-paretic one with just friction compensation. This second approach did not work either, since the gravity compensation was not considered and the overall effect induced an instability feeling in the user. In fact, during testing sessions with healthy volunteers, they complained about the effort they experienced to keep the knee and the trunk extended during the support phase, due to the extra weight of the exoskeleton. The same discomfort was reported while flexing the knee and hip joints during the swing phase.

For this reason, the third approach consisted in controlling also the non-paretic leg with an assistive mode to handle the exoskeleton weight, leaving the leg free to impose the desired trajectory. This solution provides slight assistive torques in specific gait phases to facilitate the aforementioned tasks of knee and trunk extension during stance, and joint flexion during swing that may otherwise be difficult to execute while wearing the exoskeleton. This assistive strategy might be useful to recover the physiological symmetry between sides in persons post-stroke. In fact, these subjects overuse the non-paretic limb limiting the body weight shift on the paretic limb, in order to improve stability (Lencioni et al., 2021). Therefore, in order to facilitate a more physiological gait pattern, we aimed at persuading patients to shift their body weight forward onto the paretic limb, by applying an assistive torque to the affected limb, which will consequently not collapse under body weight, thus increasing stability and keeping the patient safe.

To summarize, the focus group at T0 outlined the necessity of asymmetric control between the two legs. Specifically, it was established to have the control for the healthy leg compensating for the weight of the TWIN structure, while for the plegic leg, either full-position or assistive control could be chosen, depending on the level of impairment.

#### Observations of physiotherapists testing the system at intermediate stages (T1)

**FIGURE 2:**
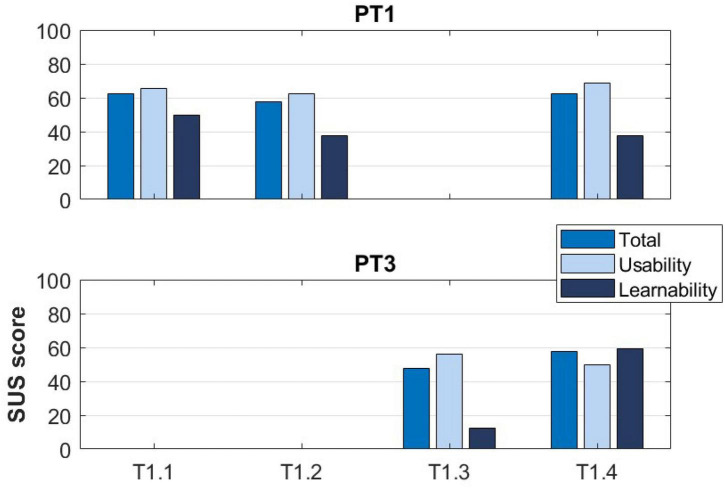
The System Usability Scale (SUS) score and subscores reported by PT1 and PT3 at T1.

During the intermediate developmental phases of TWIN-Acta, 3 physical therapists (PT1, PT2, PT3) tested the actual versions on 2–3 occasions. The assessments of usability and learnability reported by PT1 and PT3, who tested, respectively, 3 and 2 versions of TWIN-Acta, have been reported in Figure 2. All physical therapists and researchers were well experienced in working with persons with neurological disorders. All of them had the experience with technological approaches for neurorehabilitation and were familiar with robotic rehabilitation devices, virtual reality, and treadmills with augmented reality.

##### Observations and testing at T1.1

During this phase, we tested different control strategies for the two legs, i.e., the transparent control mode by applying assistance to one leg, and position-based assistance to the other leg. We tested also the use of a walker during simple over ground walking exercises.

Two physical therapists tested the device (PT1 and PT2). The SUS score and subscores reported by PT1 are displayed in Figure 2. Observations related to the testing of the TWIN exoskeleton with the transparent control mode are reported in Table 1.

**TABLE 1:** Semi-structured questionnaire responses (items 4–10) provided at T1.1 by two PTs (PT1 and PT2).

**Which aspect of the exoskeleton did you like the most and why?**
Both PTs agreed that the best aspect of the TWIN-Acta control was the possibility of varying the assistance given by the exoskeleton at each joint and separately for the two sides and that it was possible to achieve the transparency mode for one side of the exoskeleton. Moreover, one of them mentioned the fact that the assistive part could also become resistive and so possibly be used therapeutically. Further, the minor encumbrance of TWIN compared to other known exoskeletons and robots, and the fact that it allowed overground walking with regular shoes was considered positive. The PTs liked the regulation of gait parameters through the GUI available on tablets.
**Which aspect of the exoskeleton did you like the least and why?**
Neither PT liked how the exoskeleton assistance at the hip forced abduction and adduction and rotation in a non-physiological way. Furthermore, both commented on the ankle control parameters as being rigid and forcing light supination, and in general, the ankle segment was considered too rigid and without the possibility of pushing the forefoot on the ground during the push off phase of gait.
**On a scale from 1 to 7 how useful do you think the exoskeleton is useful for improving balance and gait?**
Both PTs gave a score equal to 5, indicating it was useful.
**Were the functionalities of the prototype clear and easy to learn?**
Both PTs agreed that, during the wearing of the exoskeleton, the functionalities were easy to understand and that with some practice the diverse functionalities of the exoskeleton and its control parameters would become easier to learn and consequently to adjust.
**Do you have suggestions as to how the actual prototype could be improved?**
Both PTs considered the rigidity of the ankle and its control parameters as something to improve. At foot strike, the control system induced a flexion of the knee in order to advance, this was considered a potential problem for person post-stroke that often have hypertonus around the knee in the stance phase.
**Do you think that the actual system tested could be beneficial for your patients?**
The first PT thought the system could be useful for reeducation of the correct phases of gait in various types of neurological patients, and that it could be useful for anticipating gait training in patients with muscle weakness. The second PT, along the same lines, thought that in particular persons post-stroke could benefit from the system’s facilitation of actual movement deficits and that it could induce better muscle recruitment in hip and knee flexors. The PT also thought the possibility of the system giving assist-as-needed to the movement would be beneficial for the motor learning of the patient. The need to consider altered sensibility of the leg of persons post-stroke in dressing and use of the exoskeleton was pointed out.
**Do you think your Institute/Clinic would be willing to adopt this solution in rehabilitation?**
Both therapists were convinced of their Institute’s willingness to adopt the proposed solution given the strong interest in technological solutions for rehabilitation and the fact that they could be used along more traditional approaches to increase the range of therapeutic offers provided at the Institute.

##### Observations and testing at T1.2

With respect to the previous version, in this one, the control suite was improved to allow the identification of the intention to move and to provide assistance during step initiation up to the toe-off phase. The latter was identified by considering a threshold on foot clearance from the ground. This was possible because a novel geometric model was implemented, allowing the identification of different postures. The level of assistance at each joint could be set with a precision of 1N⋅m.

The same PT (PT1) that tested the previous version of the device, performed the test also at T1.2. Table 2 collects the PT’s observations, Figure 2 reports the SUS score and subscores reported by the PT.

**TABLE 2:** Semi-structured questionnaire responses (items 4–10) provided at T1.2 by one PT (PT1).

**Which aspect of the exoskeleton did you like the most and why?**
The PT responded that with respect to the version tested before (T1.1), the system’s movements were more fluid and that the induced flexion of the knee during the terminal gait phase in the previous version of the system was no longer a problem.
**Which aspect of the exoskeleton did you like the least and why?**
The PT pointed out that the foot clearance threshold to activate the assisted modality of the system was too high.
**On a scale from 1 to 7 how useful do you think the exoskeleton is useful for improving balance and gait?**
The PT voted the usefulness of the system as 5 again.
**Were the functionalities of the prototype clear and easy to learn?**
The PT responded that the functionalities were clear and easy to learn but that at least two sessions were needed in order to learn and profit from the diverse functionalities of the system.
**Do you have suggestions as to how the actual prototype could be improved?**
The PT suggested to increase the fluidity of the system at the hip (pelvis) in order to give more control to the patient in coordinating movement of the hip with gait phases, in particular during the stance phase in which the feet were aligned.
**Do you think that the actual system tested could be beneficial for your patients?**
The PT responded that in its actual state with the actual assistive mode, the system could be very useful for patients that were more physically impaired.
**Do you think your Institute/Clinic would be willing to adopt this solution in rehabilitation?**
The PT repeated that the Institute would be interested.

##### Observations and testing at T1.3

**FIGURE 3:**
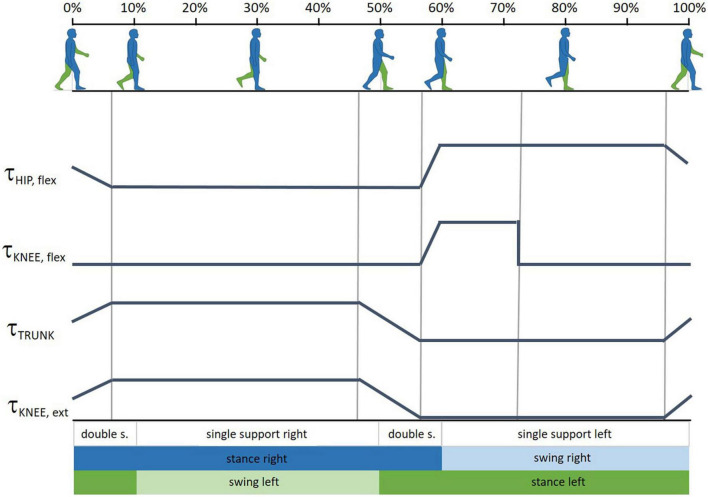
Gait cycle phases and the timing profiles of the assistance provided at articular joints at T1.3. τ_HIP,flex_ and τ_KNEE,flex_ are the assistive torque, respectively, provided at the hip and knee joint during the toe-off phase of the impaired leg; τ_TRUNK_ is the assistive torque provided to keep the torso erect; τ_KNEE,ext_ is the assistive torque provided at the impaired knee during the landing phase. Gait phase graphics modified from BoH–Own work, CC BY-SA 4.0, https://commons.wikimedia.org/w/index.php?curid=79850448.

At this stage, assistance was provided through torques applied to TWIN joints, in three ways: (i) support during toe-off phase of the impaired leg provided at the hip and knee joints (τ_HIP,flex_ and τ_KNEE, flex_ in Figure 3); (ii) support to keep the torso erect during walking (τ_TRUNK_ in Figure 3) applied during the stance phase of both legs; (iii) support of knee extension of the impaired leg during the landing phase (τ_KNEE, ext_ in Figure 3).

A novice PT (PT3) performed the test at T1.3. Table 3 collects PT’s observations and Figure 2 reports the SUS score and subscores.

**TABLE 3:** Semi-structured questionnaire responses (items 4–10) provided at T1.3 by one PT (PT3).

**Which aspect of the exoskeleton did you like the most and why?**
With respect to the previous version tested, the PT found that knee extension was more stable during the stance phase and that the control of retroversion/anteversion of the pelvis was improved.
**Which aspect of the exoskeleton did you like the least and why?**
The PT perceived an excessive length of time in the active extension of the hip at the end of the swing phase of the contralateral limb.
**On a scale from 1 to 7 how useful do you think the exoskeleton is useful for improving balance and gait?**
The PT voted the usefulness of the system as 6.
**Were the functionalities of the prototype clear and easy to learn?**
The PT again responded that the functionalities were clear and easy to learn but that at least two sessions were needed in order to learn and profit from the diverse functionalities of the system.
**Do you have suggestions as to how the actual prototype could be improved?**
The PT suggested reducing as much as possible the friction during movement so that the gait could be even more physiological and less fatiguing to the patient.
**Do you think that the actual system tested could be beneficial for your patients?**
The PT again pointed out the usefulness of the system in rehabilitation for patients that were more physically compromised.
**Do you think your Institute/Clinic would be willing to adopt this solution in rehabilitation?**
The PT stated that the Institute would be interested.

##### Observations and testing at T1.4

**FIGURE 4:**
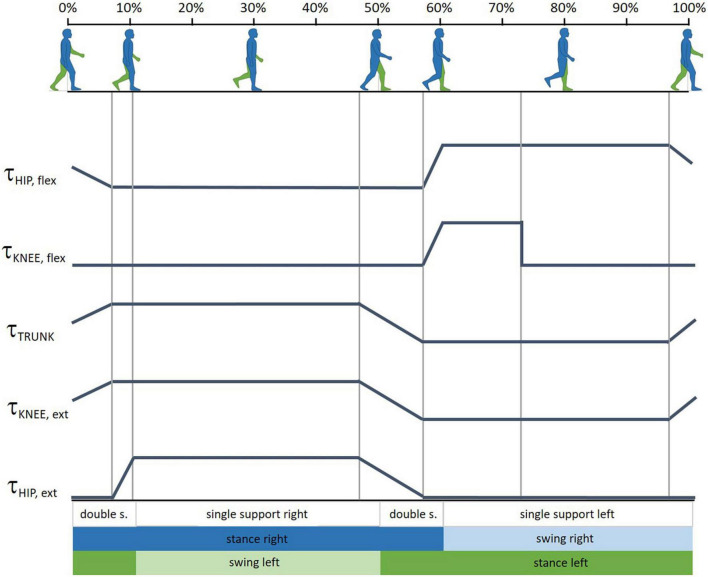
Gait cycle phases and the timing profiles of the assistance provided at articular joints at T1.4. τ_HIP,flex_ and τ_KNEE,flex_ are the assistive torque, respectively, provided at the hip and knee joint during the toe-off phase of the non-paretic leg; τ_TRUNK_ is the pelvic tilt dumping during stance phase; τ_KNEE,ext_ provides stabilization of the knee joint of the support leg; τ_HIP,ext_ helps the extension of the paretic leg during stance phase. Gait phase graphics modified from BoH–Own work, CC BY-SA 4.0, https://commons.wikimedia.org/w/index.php?curid=79850448.

At this stage, the following control features were integrated: (i) support during toe-off phase of the non-paretic leg (τ_HIP,flex_ and τ_KNEE, flex_ in Figure 4); (ii) pelvic tilt dumping during stance phase (τ_TRUNK_ in Figure 4); (iii) stabilization of the knee joint of the support leg during both stance phases (τ_KNEE, ext_ in Figure 4); and (iv) hip extension support of paretic leg during stance phase (τ_HIP, ext_ in Figure 4).

PT1 and PT3 tested the device in its T1.4 version. Table 4 collects the PTs’ observations and Figure 2 reports the SUS score and subscores.

**TABLE 4:** Semi-structured questionnaire responses (items 4–10) provided at T1.4 by PT1 and PT3.

**Which aspect of the exoskeleton did you like the most and why?**
The PTs liked the potential of the exoskeleton to provide an assist-as-needed control tailored to the patient’s needs.
**Which aspect of the exoskeleton did you like the least and why?**
The aspect the PTs liked least was the difficulty in changing walking direction while wearing the exoskeleton, although both PT considered this aspect difficult to modify given the nature of the structure.
**On a scale from 1 to 7 how useful do you think the exoskeleton is useful for improving balance and gait?**
The PTs both voted the exoskeleton’s usefulness in balance and gait rehabilitation as 5.
**Were the functionalities of the prototype clear and easy to learn?**
The PTs thought the functionality of the system was clear, but that it took time to learn to work with the system.
**Do you have suggestions as to how the actual prototype could be improved?**
There were various suggestions also based on discussions of the group during the session. The PTs suggested the possibility of an initial calibration phase, where the healthy/less affected side could be used as a base for the control parameters for the more affected side (e.g., in clearance). The PTs also suggested allowing the setting of various parameters, such as the stride length and clearance to be adapted to the patient’s capacity to voluntarily activate muscles and the importance of tailoring the assistance according to the patient’s improvement and his learning to work with the exoskeleton. Further, both PTs suggested that various control parameters could be made more automatic so that the use of the exoskeleton could be more user-friendly for the therapist. As an example, the PT3 suggested providing lumbar support, as this is needed by the majority of patients. This support could then be progressively diminished or deleted as the patient improves.
**Do you think that the actual system tested could be beneficial for your patients?**
The PTs were convinced that the system could be useful for the patients.
**Do you think your Institute/Clinic would be willing to adopt this solution in rehabilitation?**
The PTs were convinced the Institute would be interested in using the solution in rehabilitation.

## The TWIN-Acta software control

### Computing the gait phases

Following the output of session T1.4, the TWIN-Acta control suite was finally developed, as described in this section. TWIN-Acta identifies gait phases thanks to the encoders integrated into the structure of the exoskeleton, without relying on specific external sensors. In particular, the gait phases are determined by the detection of the inter-feet distance in the sagittal plane. Three possible conditions are thus possible: right foot forward, aligned feet, left foot forward. This information is then used to compute the torque provided to each joint. The TWIN-Acta control strategy is inspired by the work by Martínez et al. (2018) and relies on a state classifier that, running continuously in the background, returns the state of the exoskeleton (i.e., the real-time gait phase).

**FIGURE 5:**
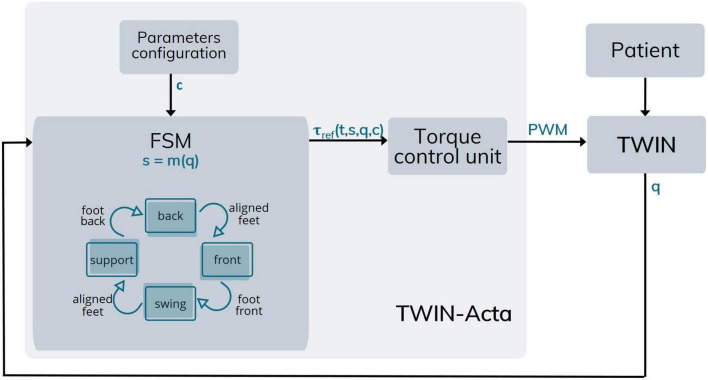
Architecture of TWIN-Acta. Following parameters configuration (c), the FSM identifies the state of the TWIN exoskeleton [s = m(q)] in which the patient is (q). Specifically, the FSM recognizes the state of the plegic leg among the four possible–represented by the blue blocks, while the arrows represent state changes of the leg–and provides the reference torque which is a function of time, exoskeleton state, position, and configuration parameters [τ_ref_ (t,s,q,c)]. The torque control unit provides the assistance (torques) to be delivered to TWIN joints [brushless DC motors controlled *via* pulse width modulation (PWM)].

The various phases of the walking cycle are handled by a Finite State Machine (FSM), which generates the signals to be sent to the active joints to provide the assistive torque. The FSM changes status according to the walking cycle, which is constantly identified thanks to the joint angles. Indeed, a “state classifier” continuously runs in the background during movements and identifies the kinematic status of the exoskeleton. The FSM, therefore, transmits the torque amplitude to be delivered to the various joints to a torque control unit, which in turn acts on the exoskeleton electric motors. The kinematic configuration of the exoskeleton, which is returned to the FSM, is obviously influenced by the contribution of the patient, who can perform free movements, assisted by the torques delivered through the various mechanical joints (Figure 5).

Unlike the state of the art methods such as those reviewed in Baud et al. (2021), the TWIN-Acta strategy does not rely on the detection of the supporting leg but on the relative position of the feet in the sagittal plane. In fact, TWIN-Acta classifies which foot has advanced the other by a certain distance, named the *relative foot distance threshold*. This parameter allows the identification of the condition of *aligned feet*, which we identified when the feet are displaced 10 cm or less along the sagittal plane. We hypothesized that foot contact with the ground occurs shortly after the change of condition, from aligned feet to right/left foot forward. For this reason, we introduced the *transition time* parameter, which indicates the time taken by the user to move their weight from one leg to the other. Conveniently, this time can be configured, based on the user’s walking speed. Once this transition time has elapsed, it is possible to assume that the foot in the rear position is that belonging to the previously supporting leg, now freed from the user’s body weight and thus preparing to take the step, becoming the swinging leg. For example, a transition from the condition of *aligned feet* to the condition of *right foot forward* indicates that the right leg becomes the supporting leg shortly after the change of state has occurred. Depending on the condition of the swinging leg or the supporting leg, the torques delivered by the motorized joints may vary. An advantage of this approach is the possibility of identifying the various gait phases with a single parameter (the inter-feet distance in the sagittal plane) without extra sensors. On the basis of this identification, command signals for controlling the motorized joints are sent to deliver assistive torques (Figure 5).

### The assist-as-needed control of TWIN-Acta

The assistive torques delivered by the motors to the joints can be set according to the level of impairment of the patient. Specifically, a set of default assistive torques is initially chosen. Then, after observing few steps of the patient using the system, the therapist may change them by tuning the control parameters, according to their subjective clinical observation. Therefore, each joint provides a different contribution, i.e., an assistive torque, for each phase of the walk. These contributions can be provided individually or in combination, i.e., it is possible to activate a single joint to deliver a specific torque, or to activate two or more joints together. Moreover, a single joint can deliver an assistive torque to accomplish more than one function, such as the joint at the hip, which is used to extend/flex the hip to perform the walk, but also to maintain the upper body of the patient in an upright position both during quiet standing and walking.

Each assistive torque delivered is the result of the analysis of the main difficulties encountered by persons post-stroke during walking and of how to address them with the exoskeleton (phases T0 and T1). These issues were addressed by providing specific assistance throughout the entire gait cycle, preferably in the form of a single assistive torque, obtained from the sum of different contributions, in particular:

• τ_KNEE, ext_: knee extension of the supporting leg;
• τ_HIP, ext_ hip extension of the supporting leg;
• τ_TRUNK_: trunk extension during the support phase;
• τ_HIP, flex_: hip flexion of the swinging leg;
• τ_KNEE, flex_: knee flexion of the swinging leg;

These contributions can be provided at the level of a single joint or in combination and, consequently, for each phase of walking, the joints of the paretic limb can rely on configurable quantities of assistance related to the patient neuromuscular deficit. This approach allows customizing the rehabilitation treatment on the patient’s deficits and, consequently, increases the probability of a physiological recovery. In addition, the ability to continuously deliver assistance throughout the entire walking cycle, makes walking safer, even in the case of possible variations in the trajectory during the different steps.

Notably, TWIN-Acta compensates for the friction of the motors and the weight of the limbs. These compensations facilitate the patient’s voluntary movements because the patient does not perceive neither the friction nor the weight of the structure as an obstacle.

As anticipated, the method aims to provide assistance only to those joints which actually require assistance and in settable quantities, depending on the specific clinical conditions of the patient, through a dedicated graphical user interface (GUI).

Regarding the supporting leg, three main components can be configured to act separately or simultaneously, according to the patient’s needs, as described below.

(1) **τ_KNEE, ext_**. A first component is the assistive torque aimed at maintaining knee extension. This torque is proportional to the knee flexion bending angle and is calculated by a proportional-derivative controller (PD) using the formula:

τ=KNEE,extp⋅kneeφ+kneed⋅kneeωknee

in which. φ_knee_ is the knee angle, equal to zero if the knee is fully extended, ω_knee_ is the angular velocity of the knee measured at each instant, p_knee_ and d_knee_ are, respectively, multiplicative parameters and a parameter to dampen any oscillations. A virtual system of the elastic damper is created at the level of the knee joint.

(2) **τ_HIP, ext_**. The second contribution is made by an assistive torque able to help hip extension. Unlike knee torque, this torque is not proportional but can be considered constant. Preferably, this torque is applied with a delay, based on the transition time, i.e., it is applied during the period that elapses between the state of aligned feet and right/left foot forward. In the case of the right foot forward, the torque will be delivered by the right hip joint, while in the case of the left foot forward, the torque will be delivered by the left hip joint.
(3) **τ_TRUNK_**. The third contribution, provided by the hip motor, is a torque helping the trunk to keep an upright position and is proportional to the tilt angle of the patient’s trunk, i.e., the angle between the longitudinal axis of the trunk and the frontal plane. This contribution helps the patient to maintain an erect posture. The tilt angle of the trunk is detected by the Inertial Measurement Unit (IMU) positioned in the pelvic assembly of the exoskeleton. Also, in this case, the amount of torque to be delivered is calculated by a PD controller on the basis of the tilt angle of the trunk:

τ=trunkp⋅hipφ+IMUd⋅hipωIMU

in which φ_IMU_ is the angle that measures the forward tilt of the trunk (equal to zero if the torso is completely erect and therefore vertical), ω_IMU_ is the speed relative to the movement of the torso, p_hip_ indicates a torque proportional to the relative angle of the torso with respect to the vertical, d_hip_ is a parameter designed to dampen any oscillations. It is possible to predict a range of values around the zero value of the tilt angle, in which no assistive torque is delivered.

As for the supporting leg, two contributions can assist the swing phase:

(1) **τ_HIP, flex_**. The first contribution relates to an assistive torque aimed at helping hip flexion, which can be considered constant. This torque is applied during almost the whole swing phase, decreasing only during the terminal swing.
(2) **τ_KNEE, flex_**. The second contribution is an assistive torque designed to help knee flexion, which is applied mainly during the initial phase of swinging the leg, to facilitate the detachment of the foot from the ground.

TWIN-Acta also plans to modify the values of the flexion torques of the hip and knee, applying compensation relative to the weight of the swinging leg. Given all the contributions described, the method allows the generation of different assistive torque profiles to be applied to the patient’s lower limbs.

## Observations of health professionals testing the system and observers of the system in use at T2

### Outcomes of health professionals testing TWIN-Acta

In all, three physical therapists (PT2, PT3, PT4), one expert neurologist (EN1), and one bioengineer (BE1) tested the system in its final version. All of them worked principally with neurological disorders, including persons post-stroke, with multiple sclerosis, and with Parkinson’s disease; the bioengineer had experience with persons post-stroke through clinical evaluations. The health professionals had from 4.5 to 12 years of experience, and the mean and standard deviation age was 38.2 and 13.5 years (range 27–60 years), one was male. All of them had previous experience with technological approaches for rehabilitation, in particular with treadmill, C-mill (Hocoma, Volketswil, Switzerland) and the Geo system (Reha Technology, Olten, Switzerland), motion analysis systems and robotic devices for the upper limb and Virtual reality systems. All of them had theoretical knowledge of exoskeletons and participated in demonstrations of exoskeletal systems for gait rehabilitation, such as HAL (Kasaoka and Sankai, 2001) and Ekso (Kolakowsky-Hayner et al., 2013).

**FIGURE 6:**
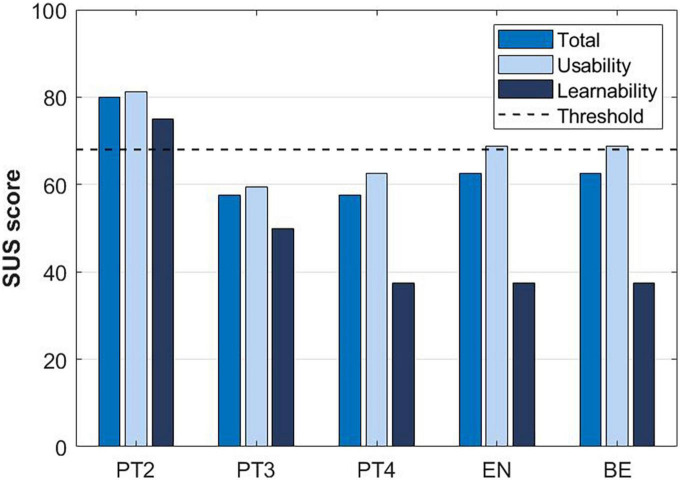
The System Usability Scale (SUS) score and subscores reported by experts in stroke rehabilitation at T2.

Subjects tested the system using both crutches and a walker. When walking with crutches in the TWIN-Acta the ipsilateral crutch is brought ahead with the leg, which is contrary to what happens in free gait where the contralateral crutch is brought ahead with the leg; this, sometimes, created confusion for the clinicians. Figure 6 reports the collected SUS scores and subscores, while Table 5 collects the health professionals’ observations.

**TABLE 5:** Semi-structured questionnaire responses (items 4–11) provided at T2 by the three physical therapists, the expert neurologist, and the bioengineer.

**Which aspect of the exoskeleton did you like the most and why?**
The possibility to selectively modulate the assistance given by the exoskeleton, depending on the affected/non-affected leg during the movement execution; The person using TWIN-Acta is able to control the speed of movement; The possibility of modulating the assistive torques separately for the stance and swing phase; The sensation during the use of TWIN-Acta of fluidity in movement both during gait and during a sit to stand movement.
**Which aspect of the exoskeleton did you like the least and why?**
The impossibility of changing the direction of walking; difficulty in weight shifting from one foot to the other during gait; a resistance from TWIN-Acta in some parts of the gait cycle; the heaviness of TWIN-Acta structure; The modification of a normal gait pattern; including also the alteration of physiological acceleration and deceleration in gait phases of a normal gait; the use of the crutches during gait with TWIN-Acta was not intuitive (*N* = 2, PT2, PT4); Having to overcome a certain resistance given by TWIN-Acta during gait, especially during knee extension; The fact that during faster gait the faster heel-strike phase made it necessary to pay attention to the phase of absorbing/loading body weight; The cost-effectiveness of rehabilitation intervention with TWIN-Acta (*N* = 1, PT2), since it needs at least one bioengineer and one physical therapist to be present during the use with a patient.
**On a scale from 1 to 7 how useful do you think the exoskeleton is useful for improving balance and gait?**
All scores were between 4 and 5 (mean 4.7 out of 7) indicating the perceived good use of the system for rehabilitation.
**Were the functionalities of the prototype clear and easy to learn?**
The system was considered easy to use and it was hypothesized that after a few training sessions with TWIN-Acta the interaction would become intuitive and the device easy to use.
**Do you have suggestions as to how the actual prototype could be improved?**
The system would need to be made lighter (*N* = 1, PT3) and give better support also when transparent control is set; In spite of TWIN-Acta being comfortable, it might be necessary to favor the smoothness of movement better; The introduction of pre-imposed testing algorithms might produce data clinically useful that would allow the monitoring of progress with rehabilitation (i.e., gait speed, distance covered during the session, the assistance used/given by each motor); The feasibility of use in clinical practice was questionable to one participant (PT4) while another (PT3) suggested that the feasibility of use in rehabilitation would be augmented by having also bigger sizes of TWIN-Acta.
**Do you think that the actual system tested could be beneficial for your patients?**
TWIN-Acta was considered by one physical therapist (PT3) as good for persons with post-stroke hemiplegia in the acute and subacute phases, with agreement from other two physical therapists that the system would be a good rehabilitation tool for persons post-stroke with a high disability level regarding gait ability and endurance. The system was further suggested to be able to provide good rehabilitation for persons with a high disability of gait resulting from multiple sclerosis or Parkinson’s Disease. Further, it was suggested that it would be very useful for paraplegic persons.
**Do you think your Institute/Clinic would be willing to adopt this solution in rehabilitation?**
The participants agreed with this sentence. This opinion was based on the technology becoming more pertinent for rehabilitation and exoskeletal/robotic systems being increasingly more user-friendly. Two participants (PT2, EN) mentioned the magnitude of resources (health personnel), with one of them (EN) thinking the use of an exoskeletal system would reduce the need for labor resources, while on the contrary, the other thought labor resource required for use of the exoskeletal system in rehabilitation could be prohibitive (PT2). The same participant (PT2) mentioned that the high cost of the system might be a problem.
**Further suggestions**
One participant (PT4) pointed out the need for a lighter system and an “easiness” in the use and set up of the system in order for therapy to be semiautonomous so that patients can use it for longer periods for enhanced therapeutic effect. A second participant (PT2) pointed out that a bigger range of shoe sizes than that provided by the present system was necessary.

*N* indicates the number of experts reporting a specific comment; if *N* is not specified it means that the consideration was shared among all the respondents.

### Outcomes from the focus group observing the TWIN-Acta being used at the T2 testing session

The focus group that observed TWIN-Acta while in action, was composed of clinicians having vast experience working with persons affected by neurological disorders and therapy based on robots, treadmill, and telerehabilitation. They all claimed only theoretical knowledge of exoskeletons and thought the observed prototype could be applied to the rehabilitation of gait in various patient groups. Table 6 collects their observations.

**TABLE 6:** Semi-structured questionnaire responses (items 4–11) provided at T2 by the focus group of four external clinical observers.

**Which aspect of the exoskeleton did you like the most and why?**
The exoskeleton aspects they liked most were the possibility of control: the regulation of the support given to the hip and knee and in continuing a movement initiated by the patient. Further, it was mentioned that gait rehabilitation time could be shortened.
**Which aspect of the exoskeleton did you like the least and why?**
What they liked least were the altered postural control and balance that rendered it necessary to use a walker, as well as, the need for two persons during a rehabilitation session.
**On a scale from 1 to 7 how useful do you think the exoskeleton is useful for improving balance and gait?**
The Focus group clinicians voted TWIN-Acta solution 5/7 in potential usefulness in improving balance and gait indicating well perceived usefulness of the system in improving balance and gait.
**Were the functionalities of the prototype clear and easy to learn?**
Clarity of the prototype functionalities and learnability was considered good but it necessitated training to use.
**Do you have suggestions as to how the actual prototype could be improved?**
The focus group suggested improvement of the system’s balance aspects that would thus eliminate the need for the support/walker. Further, they suggested the anatomical limits/measures be enlarged. They also suggested that the system could be improved by an analysis of muscle activity at the end of each session giving information on how much the patients were recruiting their muscles with respect to the assistive forces from the exoskeleton, and a trend line of the same information as rehabilitation progresses.
**Do you think that the actual system tested could be beneficial for your patients?**
Regarding whether the solution tested might be good for their patients, the focus group thought the solution tested was optimal for patients in subacute post-stroke phase that do not have good balance reactions, nor good propulsion ability (early in their recovery).
**Do you think your Institute/Clinic would be willing to adopt this solution in rehabilitation?**
The Focus group thought their Institute would willingly adopt the solution for the rehabilitation of gait. However, cost, facility of use, and duration of the rehabilitation session were suggested to be important issues.
**Further suggestions**
They suggested improvement of the prototype could include balance and weight-shifting control in order to evaluate its effect on motor module activations.

### Outcomes of persons post-stroke testing TWIN-Acta

The enrolled persons post-stroke (PPS01, PPS02, PPS03, PPS03, PPS04, and PPS05) tested the final version of TWIN-Acta using a walker. Table 7 reports the collected SUS scores and subscores and the SRMS scale. The averaged SUS indicated that the system was acceptable (mean ± SD, 68.0 ± 21.0), with good usability (73.8 ± 23.7) and lower learnability (45.0 ± 14.3). The 7-item SRMS score was greater than the cut-off score for all participants post-stroke, indicating a high level of motivation (mean ± SD, 26.2 ± 3.3).

**TABLE 7:** SUS scale and its subscores and SRMS scale reported from person post-stroke which tested the final version of TWIN-Acta (T2).

Subjects	Paretic side	SUS_Learnability_	SUS_Usability_	SUS_Total_	SRMI
PPS01	LX	25.0	59.4	52.5	26
PPS02	RX	50.0	81.3	75.0	27
PPS03	LX	62.5	87.5	82,5	30
PPS04	LX	37.5	40.6	40.0	21
PPS05	RX	50.0	100.0	90.0	27

SUS, System Usability Scale; SRMS, Stroke Rehabilitation Motivation Scale; LX, left; RX, right.

## Discussion and future work

This study describes the user-centered design and development and the initial validation of the TWIN-Acta control suite to be adopted in gait rehabilitation of persons post-stroke using the TWIN-powered lower limb exoskeleton, originally designed for spinal cord injured persons. The rehabilitation goal is to restore as much as possible the physiological gait pattern with assist-as-needed provided by the exoskeleton to reduce existent post-stroke motor deficiencies. For this purpose, an interdisciplinary group of engineers and clinicians collaborated along the developmental process of TWIN-Acta to define the design specifications for the adaptation of the control modality in accordance with user needs. This collaboration led to the development of the TWIN-Acta control suite, which provides assist-as-needed and enables tailored support based on the residual ability of the person post-stroke. Health professionals observing and testing the TWIN-Acta along its developmental phases were overall positive in their ratings of its usefulness and learnability, and in particular, the possibility of modulating the assistance given by the exoskeleton during the movement execution was considered important. They also agreed that the TWIN-Acta would be useful in gait rehabilitation for persons post-stroke and that their Institution would probably be willing to employ the proposed rehabilitation solution.

### Defining the objectives and technical solutions

Restoring gait, both in quantity and quality, for persons post-stroke is a prime objective in their rehabilitation. In the first exploratory phase carried out in close collaboration between engineers and clinicians, the physiological and rehabilitation needs of users were identified (Chadran et al., 2020). An important goal for therapeutic exoskeletons is to help patients achieve outcomes that are otherwise difficult under their own strengths and so the main principle was the assist-as-needed control strategy. The user interface is similarly an important issue in robotic exoskeleton development, indeed intuitive and flexible user interfaces are essential for ease of use and successful rehabilitation (Young and Ferris, 2016).

An important issue found early in the developmental phase of TWIN-Acta was how to account for the different needs of the paretic and non-paretic leg of a person with hemiplegia. The first idea of an exoskeleton with only one leg mounted was immediately abolished since it created a balancing issue. Compensating only for friction was found not to be acceptable while adding assistance to knee and hip flexion of the non-paretic side was found an acceptable solution to clinical end users, as it allowed to compensate for the weight of the exoskeleton, left the leg free to impose the desired trajectory and increased the symmetry of effort between sides. In this way, there was free interaction in establishing the whole gait pattern between the exoskeleton and the wearer, with a variable level of joint assistance across joints and sides. This configuration is suitable for different individuals with residual voluntary control and is consistent with the recommendation of an assist-as-needed approach for maximizing motor learning for persons with neurological disorders (Durandau et al., 2019). This is also appropriate for an exoskeleton intended for rehabilitation, allowing the adjustment of the control system to the individual improvement in exoskeleton-wearer interaction and in motor function (Young and Ferris, 2016).

Another issue we had to face early in the developmental phase was the support to provide to the person post-stroke during overground walking with the exoskeleton. While some studies of overground robotic exoskeletons have chosen to use unilateral upper limb support for persons post-stroke (Nolan et al., 2020), in this study we used a high walker providing bilateral support. The use of the walker with the exoskeleton allowed us to evade the problem of asymmetric support by persons post-stroke and maintain a symmetry essential to gait training and recovery.

### Validation of technical solutions and evaluation of TWIN-Acta by health professionals and persons post-stroke

TWIN-Acta is needed to meet the clinical end user needs in terms of functionality, usability, and rehabilitation applicability to persons with stroke, in order to identify users’ expectations and needs for the intended application. This user-centered approach is in line with the recommendation for holistic evaluation of technological devices intended for rehabilitation with close collaboration between technical and clinical partners (Armannsdottir et al., 2020; Torricelli et al., 2020). The whole evolution of TWIN-Acta included an expert focus group, a formative phase based on the international standard IEC 62366-1:2015 (Scherer and Gouveia Filho, 2019) in order to progressively improve the exoskeleton for rehabilitation of persons post-stroke, and a final testing phase of the ultimate TWIN-Acta control solution. The protocol included standardized questionnaires on perceived (subjective) usability and semistructured interviews in line with recommendations (Simonsen and Hertzum, 2010).

The subjective experience of wearing the exoskeleton and observing it in use by others is essential for forming an impression and understanding the potential of the exoskeleton in rehabilitation (Meyer et al., 2019). This was implemented at the initial stage with the involvement of expert clinicians and engineers and during the iterative stages of the control systems’ development with clinicians, which were actual end users in terms of being the prescribers and actually carrying out rehabilitation of persons post-stroke. This allowed us to have the longitudinal observation of users on usability aspects of the initial and further adapted version of TWIN-Acta.

The main areas of the user centric aspect investigated were user acceptance of the exoskeleton, including perceived usefulness, and willingness to make further use of the exoskeleton. In this study, all the clinic end user participants had previous experience in using technology for the rehabilitation of neurological disorders, hence technology acceptance was not an issue. This is of paramount importance since, without the health professionals’ willingness to use exoskeletons regularly in rehabilitation, the health-promoting potential of such devices could not be established.

Effort and outcome are key factors in the use of technology for rehabilitation, therefore assessing the usability and usefulness of TWIN-Acta perceived by clinicians was instrumental in finding the best design solutions. These factors include time to put on the exoskeleton, the comfort of wearing it, and the perceived usefulness of applying it to gait rehabilitation and to directly influence the intention to use the solution (Hill et al., 2017; Weber and Stein, 2018). Perceived exertion, wearing comfort, perceived usefulness, and adjustability are aspects that have already been tested, with positive ratings, on the TWIN system as applied to persons with spinal cord injuries (Laffranchi et al., 2021). In the present study, factors considered positive of TWIN-Acta throughout the evolution phases included the provision of assistance-as-needed and the fact that it could provide new possibilities for rehabilitation of gait and balance in more disabled persons post-stroke, while negative aspects included the weight of the structure and the difficulty in changing the direction of gait.

The usability and learnability aspects of the solutions indicated an increasingly more refined modular control in the assistance-as-needed settings of TWIN-Acta during the developmental phase, and an achieved feeling of symmetry between sides with respect to the effort needed to move the exoskeleton. The overall evaluation of the combined usability and learnability was mostly acceptable. Usefulness in gait and balance rehabilitation was consistently rated between 5 and 6 out of 7 during the developmental phase. In general, the functionalities of the system were considered clear and it was thought that the interaction with the system would become easy with training. During the final validation of the system by clinical testers and an observing focus group, the overall rate was again around 5 out of 7, confirming that health professionals considered it useful in the rehabilitation of persons post-stroke. There was some controversy about the TWIN-Acta’s usefulness and learnability according to the SUS scale of clinical experts, in that scores ranged from 40 to 80, with learnability scores being lower than usability scores. The fact that learnability did not improve between T1 and T2 is due to the complexity of the TWIN-Acta control, which increased during the developmental stages.

Regarding the final version T2 as tested by persons post-stroke, the overall SUS indicated that they evaluated the system as acceptable, with excellent usability (>75), however, they also confirmed the low learnability of the system. Moreover, these results may be also linked to the present testing protocol design, which required subjects to use the device only in one session, a prolonged use of the exoskeleton would probably have improved the learnability aspect of the system. In spite of this controversy, all clinical end users had the belief that TWIN-Acta would be useful in the field of rehabilitation for neurological disorders. Kozlowski et al. (2015) found that the average number of sessions to learn to walk properly wearing an exoskeleton in SCI patients was 15 sessions and that this high number of sessions was required as learning to walk with an exoskeleton needed not only motor but also mental effort. This aspect must be taken into consideration in future rehabilitation studies with persons post-stroke, as in the present user-centered developmental study, the main focus was on the clinical view and usability perception of health professionals. Nonetheless, the preliminary data on the motivation of persons post-stroke (i.e., high SRMS scores) are promising.

### The use of TWIN-Acta for post-stroke rehabilitation

The user centered developmental phase was an iterative process with the end users’ perspectives suggesting priorities for successful use in rehabilitation. The tight collaboration between expert engineers, clinical researchers, and health professionals is certainly a strength of the present study. This collaboration, carried out throughout the evolution of the TWIN-Acta control system, has allowed the identification of the most important features and the tailored technical solutions that an exoskeleton must implement to be useful in the gait rehabilitation of persons post-stroke. This user-centered approach, with health professionals actually involved in the rehabilitation process of persons with neurological disorders playing a central role in the judgment of proposed solutions and further evolution of the exoskeleton, was similarly key in arriving at the final TWIN-Acta system, well accepted by persons post-stroke. The use of the TWIN-Acta control suite allows a mix between an assistive and a therapeutic exoskeleton. This bifunctionality of TWIN-Acta can make the exoskeleton helpful both as a therapeutic device in gait rehabilitation, as well as, to increase the current physical capability of the user while wearing it (Maeshima et al., 2011; Nilsson et al., 2014).

With respect to other exoskeletons, we do not evaluate the gait phase with special sensors to promote a rhythmic walk. One of the most advantageous aspects of our approach is the use of a single parameter for identifying the walking phase, i.e., the distance between the feet with respect to the sagittal plane. A simple approach to walking analysis is thus obtained, which is sufficient to describe gait in an exhaustive manner and to evaluate the correct assistance torque to be provided at the joints. Other groups have implemented predictive strategies to regulate step parameters before the actual movement (Martinez-Hernandez and Dehghani-Sanij, 2018; Sahoo et al., 2020; Chen et al., 2021; Zhang et al., 2021). But although promising, these solutions require the use of additional sensors and the implementation of trajectory-based walking and were thus here discarded in favor of a time-independent strategy. Since only one parameter is used to identify the gait phases, one of the main advantages of the TWIN-Acta is the avoidance of incorrect detections of the walking phase. Another advantage is that the method does not require any additional sensors other than the encoders already present in the joints. This greatly simplifies the control scheme and reduces the hardware complexity of the exoskeleton.

Overall, the TWIN-Acta constitutes a promising tool for post-stroke rehabilitation. Assessment of motor recovery following rehabilitation with the TWIN-Acta will be the object of future work. Also, novel strategies for maximization of user engagement will be explored, such as brain-driven control (Paek et al., 2021).

### Limitations

The main limitations of the present study are the small number of professionals and persons post-stroke involved in the feasibility tests of the TWIN-Acta control suite. Moreover, our analysis focused on subjective metrics to assess the usability and learnability of the system. Instead, future work will make use of objective metrics, such as the calculation of gait phases and relative parameters (e.g., % double support time, stride length, gait speed) and muscular activation patterns (e.g., muscle synergies, EMG-based co-contraction indexes).

Limitations of the exoskeleton itself include the fact that TWIN at the moment requires at least two persons assisting during rehabilitation sessions which makes it costly in terms of personnel effort. Yet, another limitation is the weight of the exoskeleton, along with the restriction in size of persons that could use the exoskeleton, which implies that very tall or somewhat overweight persons may not fit in it. Further, while the fluidity of the movement was considered much better in the final validation, however, this is still considered a factor to improve. Finally, the difficulty in changing direction could certainly create difficulty in smaller rehabilitative environments.

## Conclusion

In its present form, the TWIN-Acta control system is a personalized neurorehabilitation technology that enables exoskeletons to physically interact with persons with neuromotor disabilities to maximize the recovery of compromised gait and balance. The final solution was found acceptable to clinical end-users and considered appropriate as a rehabilitation tool for persons post-stroke, further, all agreed that TWIN-Acta would be of interest to be adopted by their rehabilitation institute.

## Data availability statement

The raw data supporting the conclusions of this article will be made available by the authors, without undue reservation.

## Ethics statement

The studies involving human participants were reviewed and approved by Comitato Etico Fondazione Don Carlo Gnocchi, Milan, Italy (21 June 2018). The patients/participants provided their written informed consent to participate in this study.

## Author contributions

MS, TL, ML, MiC, MF, LD, and JJ conceived the study. WH, CV, SS, and SM developed the TWIN-Acta control suite. JJ developed the semi-structured questionnaire. MS, TL, and JJ wrote the first draft of the manuscript. MS and TL prepared the figures. MS, TL, CV, SS, SM, MF, and JJ participated to the tests at T2. All authors participated to the focus group at T0 and to the tests at T1, read, and approved the final version of the manuscript.

## References

[B1] Armannsdottir A. L. Beckerle P. Moreno J. C. van Asseldonk E. H. F. Manrique-Sancho M. T. Del-Ama A. J. (2020). Assessing the involvement of users during development of lower limb wearable robotic exoskeletons: A survey study. *Hum. Factors* 62 351–364. 10.1177/0018720819883500 31928418 PMC7221858

[B2] Bangor A. Kortum P. T. Miller J. T. (2009). Determining what individual SUS scores mean: Adding an adjective rating scale. *J. Usability Stud. Arch.* 4 114–123.

[B3] Baud R. Manzoori A. R. Ijspeert A. Bouri M. (2021). Review of control strategies for lower-limb exoskeletons to assist gait. *J. Neuroeng. Rehabil.* 18:119. 10.1186/s12984-021-00906-3 34315499 PMC8314580

[B4] Bernhardt J. Hayward K. S. Kwakkel G. Ward N. S. Wolf S. L. Borschmann K. (2017). Agreed definitions and a shared vision for new standards in stroke recovery research: The stroke recovery and rehabilitation roundtable taskforce. *Neurorehabil. Neural Repair* 31 793–799. 10.1177/1545968317732668 28934920

[B5] Billinger S. A. Arena R. Bernhardt J. Eng J. J. Franklin B. A. Johnson C. M. (2014). Physical activity and exercise recommendations for stroke survivors: A statement for healthcare professionals from the American Heart Association/American Stroke Association. *Stroke* 45 2532–2553. 10.1161/STR.0000000000000022 24846875

[B6] Borsci S. Federici S. Lauriola M. (2009). On the dimensionality of the System Usability Scale: A test of alternative measurement models. *Cogn. Process.* 10 193–197. 10.1007/s10339-009-0268-9 19565283

[B7] Brooke J. (1996). “SUS-A quick and dirty usability scale (in Usability Evaluation in Industry,” in *Usability evaluation in industry*, eds Jordan P. W. Thomas B. McLelland I. Weerdmeester B. A. (Boca Raton, FL: CRC Press).

[B8] Calabrò R. S. Naro A. Filoni S. Pullia M. Billeri L. Tomasello P. (2019). Walking to your right music: A randomized controlled trial on the novel use of treadmill plus music in Parkinson’s disease. *J. Neuroeng. Rehabil.* 16:68. 10.1186/s12984-019-0533-9 31174570 PMC6555981

[B9] Calabrò R. S. Naro A. Russo M. Bramanti P. Carioti L. Balletta T. (2018). Shaping neuroplasticity by using powered exoskeletons in patients with stroke: A randomized clinical trial. *J. Neuroeng. Rehabil.* 15:35. 10.1186/s12984-018-0377-8 29695280 PMC5918557

[B10] Campbell S. M. Diduch C. P. Sensinger J. W. (2020). Autonomous assistance-as-needed control of a lower limb exoskeleton with guaranteed stability. *IEEE Access* 8 51168–51178. 10.1109/ACCESS.2020.2973373

[B11] Chadran S. Al-Sa’di A. Ahmad E. (2020). Exploring user centered design in healthcare: A literature review. *Paper presented at the 2020 4th international symposium on multidisciplinary studies and innovative technologies (ISMSIT)*, Istanbul. 10.1109/ISMSIT50672.2020.9255313

[B12] Chen X. Zhang K. Liu H. Leng Y. Fu C. (2021). A probability distribution model-based approach for foot placement prediction in the early swing phase with a wearable IMU sensor. *IEEE Trans. Neural Syst. Rehabil. Eng.* 29 2595–2604. 10.1109/TNSRE.2021.3133656 34874865

[B13] Doyle S. Bennett S. Fasoli S. E. McKenna K. T. (2010). Interventions for sensory impairment in the upper limb after stroke. *Cochrane Database Syst. Rev.* 2010:Cd006331. 10.1002/14651858.CD006331.pub2 PMC6464855 20556766

[B14] Durandau G. Farina D. Asin-Prieto G. Dimbwadyo-Terrer I. Lerma-Lara S. Pons J. L. (2019). Voluntary control of wearable robotic exoskeletons by patients with paresis via neuromechanical modeling. *J. Neuroeng. Rehabil.* 16:91. 10.1186/s12984-019-0559-z 31315633 PMC6637518

[B15] Ferrarin M. Rabuffetti M. Geda E. Sirolli S. Marzegan A. Bruno V. (2018). Influence of the amount of body weight support on lower limb joints’ kinematics during treadmill walking at different gait speeds: Reference data on healthy adults to define trajectories for robot assistance. *Proc. Inst. Mech. Eng. H* 232 619–627. 10.1177/0954411918776682 29890931

[B16] Hatem S. M. Saussez G. Della Faille M. Prist V. Zhang X. Dispa D. (2016). Rehabilitation of motor function after stroke: A multiple systematic review focused on techniques to stimulate upper extremity recovery. *Front. Hum. Neurosci.* 10:442. 10.3389/fnhum.2016.00442 27679565 PMC5020059

[B17] Hidayah R. Bishop L. Jin X. Chamarthy S. Stein J. Agrawal S. K. (2020). Gait adaptation using a cable-driven active leg exoskeleton (C-ALEX) with post-stroke participants. *IEEE Trans. Neural Syst. Rehabil. Eng.* 28 1984–1993. 10.1109/TNSRE.2020.3009317 32746320

[B18] Hill D. Holloway C. S. Ramirez D. Z. M. Smitham P. Pappas Y. (2017). What are user perspectives of exoskeleton technology? A literature review. *Int. J. Technol. Assess. Health Care* 33 160–167. 10.1017/S0266462317000460 28849760

[B19] Hsu C. Y. Cheng Y. H. Lai C. H. Lin Y. N. (2020). Clinical non-superiority of technology-assisted gait training with body weight support in patients with subacute stroke: A meta-analysis. *Ann. Phys. Rehabil. Med.* 63 535–542. 10.1016/j.rehab.2019.09.009 31676456

[B20] Johnson W. Onuma O. Owolabi M. Sachdev S. (2016). Stroke: A global response is needed. *Bull. World Health Organ.* 94 634–634A. 10.2471/BLT.16.181636 27708464 PMC5034645

[B21] Kasaoka K. Sankai Y. (2001). “Predictive control estimating operator’s intention for stepping-up motion by exoskeleton type power assist system HAL,” in *Proceedings of the 2001 IEEE/RSJ international conference on intelligent robots and systems. expanding the societal role of robotics in the next millennium (Cat. No.01CH37180)*, (Maui, HI: IEEE).

[B22] Kolakowsky-Hayner S. A. Crew J. Moran S. Shah A. (2013). Safety and feasibility of using the EksoTM bionic exoskeleton to aid ambulation after spinal cord injury. *J. Spine* 4 1–8. 10.4172/2165-7939.S4-003

[B23] Kozlowski A. Bryce T. Dijkers M. (2015). Time and effort required by persons with spinal cord injury to learn to use a powered exoskeleton for assisted walking. *Top. Spinal Cord Inj. Rehabil.* 21 110–121. 10.1310/sci2102-110 26364280 PMC4568092

[B24] Krucoff M. O. Rahimpour S. Slutzky M. W. Edgerton V. R. Turner D. A. (2016). Enhancing nervous system recovery through neurobiologics, neural interface training, and neurorehabilitation. *Front. Neurosci.* 10:584. 10.3389/fnins.2016.00584 28082858 PMC5186786

[B25] Laffranchi M. D’Angella S. Vassallo C. Piezzo C. Canepa M. De Giuseppe S. (2021). User-centered design and development of the modular TWIN lower limb exoskeleton. *Front. Neurorobot.* 15:709731. 10.3389/fnbot.2021.709731 34690732 PMC8529015

[B26] Lencioni T. Anastasi D. Carpinella I. Castagna A. Crippa A. Gervasoni E. (2021). Strategies for maintaining dynamic balance in persons with neurological disorders during overground walking. *Proc. Inst. Mech. Eng. H* 235 1079–1087. 10.1177/09544119211023624 34112028

[B27] Maeshima S. Osawa A. Nishio D. Hirano Y. Takeda K. Kigawa H. (2011). Efficacy of a hybrid assistive limb in post-stroke hemiplegic patients: A preliminary report. *BMC Neurol.* 11:116. 10.1186/1471-2377-11-116 21943320 PMC3198922

[B28] Martínez A. Lawson B. Goldfarb M. (2018). “A velocity-based flow field control approach for reshaping movement of stroke-impaired individuals with a lower-limb exoskeleton,” in *Proceedings of the 2018 40th annual international conference of the IEEE engineering in medicine and biology society (EMBC)*, (Honolulu, HI: IEEE). 10.1109/EMBC.2018.8512807 30440982

[B29] Martinez-Hernandez U. Dehghani-Sanij A. A. (2018). Adaptive Bayesian inference system for recognition of walking activities and prediction of gait events using wearable sensors. *Neural Netw.* 102 107–119. 10.1016/j.neunet.2018.02.017 29567532

[B30] Mehrholz J. Pohl M. (2012). Electromechanical-assisted gait training after stroke: A systematic review comparing end-effector and exoskeleton devices. *J. Rehabil. Med.* 44 193–199. 10.2340/16501977-0943 22378603

[B31] Mehrholz J. Thomas S. Werner C. Kugler J. Pohl M. Elsner B. (2017). Electromechanical-assisted training for walking after stroke. *Cochrane Database Syst. Rev.* 5:CD006185. 10.1002/14651858.CD006185.pub4 PMC6481755 28488268

[B32] Meyer J. T. Schrade S. O. Lambercy O. Gassert R. (2019). “User-centered design and evaluation of physical interfaces for an exoskeleton for paraplegic users,” in *Proceedings of the 2019 IEEE 16th international conference on rehabilitation robotics (ICORR)* (Toronto, ON: IEEE), 1159–1166. 10.1109/ICORR.2019.8779527 31374786

[B33] Mihelj M. Nef T. Riener R. (2007). A novel paradigm for patient-cooperative control of upper-limb rehabilitation robots. *Adv. Robot.* 21 843–867. 10.1163/156855307780851975

[B34] Morone G. Paolucci S. Cherubini A. De Angelis D. Venturiero V. Coiro P. (2017). Robot-assisted gait training for stroke patients: Current state of the art and perspectives of robotics. *Neuropsychiatr. Dis. Treat.* 13 1303–1311. 10.2147/NDT.S114102 28553117 PMC5440028

[B35] Murray S. A. Ha K. H. Hartigan C. Goldfarb M. (2014). An assistive control approach for a lower-limb exoskeleton to facilitate recovery of walking following stroke. *IEEE Trans. Neural Syst. Rehabil. Eng.* 23 441–449. 10.1109/TNSRE.2014.2346193 25134084

[B36] Nilsson A. Vreede K. S. Haglund V. Kawamoto H. Sankai Y. Borg J. (2014). Gait training early after stroke with a new exoskeleton–the hybrid assistive limb: A study of safety and feasibility. *J. Neuroeng. Rehabil.* 11:92. 10.1186/1743-0003-11-92 24890413 PMC4065313

[B37] Nolan K. J. Karunakaran K. K. Chervin K. Monfett M. R. Bapineedu R. K. Jasey N. N. (2020). Robotic exoskeleton gait training during acute stroke inpatient rehabilitation. *Front. Neurorobot.* 14:581815. 10.3389/fnbot.2020.581815 33192438 PMC7661791

[B38] Paek A. Y. Brantley J. A. Ravindran A. S. Nathan K. He Y. Eguren D. (2021). A roadmap towards standards for neurally controlled end effectors. *IEEE Open J. Eng. Med. Biol.* 2 84–90. 10.1109/OJEMB.2021.3059161 35402986 PMC8979628

[B39] Rosenblatt N. J. Grabiner M. D. (2010). Measures of frontal plane stability during treadmill and overground walking. *Gait Posture* 31 380–384. 10.1016/j.gaitpost.2010.01.002 20129786

[B40] Sahoo S. Panda S. K. Pratihar D. K. Mukhopadhyay S. (2020). Prediction of step length using neuro-fuzzy approach suitable for prosthesis control. *IEEE Trans. Instrum. Meas.* 69 5658–5665.

[B41] Sauro J. (2011). *A practical guide to the system usability scale: Background, benchmarks and best practices.* Denver, CO: CreateSpace.

[B42] Scherer D. Gouveia Filho F. F. (2019). “Documentation template for the usability engineering process for medical devices,” in *World congress on medical physics and biomedical engineering 2018*, eds Lhotska L. Sukupova L. Lacković I. Ibbott G. (Singapore: Springer). 10.1016/j.mri.2021.11.014

[B43] Sczesny-Kaiser M. Trost R. Aach M. Schildhauer T. A. Schwenkreis P. Tegenthoff M. (2019). A randomized and controlled crossover study investigating the improvement of walking and posture functions in chronic stroke patients using HAL exoskeleton - The HALESTRO study (HAL-Exoskeleton STROke Study). *Front. Neurosci.* 13:259. 10.3389/fnins.2019.00259 30983953 PMC6450263

[B44] Simonsen J. Hertzum M. (2010). “Iterative participatory design,” in *Design research: Synergies from interdisciplinary perspectives*, Vol. 1 eds Simonsen J. Bærenholdt J. O. Büscher M. Scheuer J. D. (London: Routledge), 16–32. 10.4324/9780203855836

[B45] Torricelli D. Rodriguez-Guerrero C. Veneman J. F. Crea S. Briem K. Lenggenhager B. (2020). Benchmarking wearable robots: Challenges and recommendations from functional, user experience, and methodological perspectives. *Front. Robot. AI* 7:561774. 10.3389/frobt.2020.561774 33501326 PMC7805816

[B46] van Hedel H. J. Tomatis L. Muller R. (2006). Modulation of leg muscle activity and gait kinematics by walking speed and bodyweight unloading. *Gait Posture* 24 35–45. 10.1016/j.gaitpost.2005.06.015 16099161

[B47] Vassallo C. DeGiuseppe S. Piezzo C. Maludrottu S. Cerruti G. D’Angelo M. L. (2020). “Gait patterns generation based on basis functions interpolation for the TWIN lower-limb exoskeleton,” in *Proceedings of the 2020 IEEE international conference on robotics and automation (ICRA)*, (Paris: IEEE). 10.1109/ICRA40945.2020.9197250

[B48] Weber L. M. Stein J. (2018). The use of robots in stroke rehabilitation: A narrative review. *Neurorehabilitation* 43 99–110. 10.3233/NRE-172408 30056437

[B49] White G. Cordato D. O’Rourke F. Mendis R. Ghia D. Chan D. (2012). Validation of the stroke rehabilitation motivation scale: A pilot study. *Asian J. Gerontol. Geriatr.* 7 80–87.

[B50] Young A. J. Ferris D. P. (2016). State of the art and future directions for lower limb robotic exoskeletons. *IEEE Trans. Neural Syst. Rehabil. Eng.* 25 171–182. 10.1109/TNSRE.2016.2521160 26829794

[B51] Zhang K. Liu H. Fan Z. Chen X. Leng Y. de Silva C. W. (2021). Foot placement prediction for assistive walking by fusing sequential 3D gaze and environmental context. *IEEE Robot. Autom. Lett.* 6 2509–2516. 10.1109/LRA.2021.3062003

